# Editorial: Pharmacogenetics of psychiatric disorders

**DOI:** 10.3389/fgene.2024.1523071

**Published:** 2025-01-09

**Authors:** Sarah Allegra, Silvia De Francia, Stefano Comità, Maria Grazia Morgese, Thomas M. Polasek

**Affiliations:** ^1^ Department of Clinical and Biological Sciences, University of Turin, San Luigi Gonzaga University Hospital, Turin, Italy; ^2^ Department of Clinical and Experimental Medicine, University of Foggia, Foggia, Italy; ^3^ Centre for Medicines Use and Safety, Monash University, Melbourne, VIC, Australia

**Keywords:** pharmacogenomic (PGx) research, pharmacogenomic, antidepresants, major depressive disorder, cocaine, drug abuse, prescribing, personalized and precision medicine

Psychotropic medications are the gold-standard for treating many different psychiatric disorders. Nevertheless, interindividual differences in the efficacy and adverse effects of these medications is a major challenge in clinical practice (Smoller, 2014). This variability can be attributed to the influence of genetic and non-genetic factors, but it is increasingly understood how genetics plays an important role in some cases (Maj et al., 2020; Maj et al., 2021; Salazar de Pablo et al., 2021). Pharmacogenomics (PGx) is the study of variability in medication response on a hereditary basis (Polasek, 2024). This field of study has taken-off in psychiatry because many psychotropic medications are metabolically cleared by polymorphic enzymes such as CYP2D6 (Lohoff and Ferraro, 2010; Muller, 2020). A genetic component can contribute to intolerable adverse effects via impaired metabolism, often leading to scarce medication adherence (Biso et al., 2024). Conversely, despite taking treatment, some individuals do not exhibit a sufficient clinical improvement, sometimes via increased metabolism causing sub-therapeutic concentrations. These situations are classified as PGx via pharmacokinetic changes (Polasek, 2024). Variation in the genes encoding proteins that directly determine pharmacodynamics also occurs (e.g., molecular targets), and this type of PGx is now receiving increased attention, although the clinical importance of these genes is still poorly understood compared with those affecting pharmacokinetics (Mostafa et al., 2022; Polasek and Peck, 2024). Despite many challenges, PGx testing with psychotropic medications is beginning to allow for more personalized prescribing (Pirmohamed, 2023; Polasek et al., 2019). In this Research Topic, four interesting manuscripts are presented – three that are concerned with the clinical implementation of PGx in psychiatry practice (Barlati et al.; Polasek; Tesfamicael et al.) and one that demonstrates the complexities of the underlying molecular mechanisms involved in treating drug abuse and dependence, in this case, L-methionine for cocaine addiction (Wang et al.).

Pharmacogenomics can be used “pre-emptively” and “reactively” in relation to prescribing, to avoid future patient harm from medications and to diagnose medication-related problems, respectively (Polasek). The PGx benefit score (PGxBS) was proposed as a way to quantitatively measure the congruency between medication-related problems and actionable genotypes with high levels of clinical evidence according to the clinical pharmacogenomics implementation consortium guidelines (CPIC). Polasek described a structured approach to PGx consulting in medical practice, consisting of the following steps: listing current and past medications, determining the availability of PGx guidelines for each medication, assessing adequacy of therapeutic trials (dose and duration of treatment), determining therapeutic outcomes (intolerable adverse effects and/or unexplained poor efficacy), deciding on the congruency between therapeutic outcomes and the PGx tests results, and finally, calculating the patient’s PGxBS. Positive scores indicate congruency between PGx and therapeutic outcomes, whereas negative scores mean incongruency, suggesting that PGx has been unhelpful in explaining the patient’s medication-related problems. Since this paper introduces the PGxBS for the first time, further work is required to determine its clinical utility.

Through a systematic review and an updated meta-analysis, Tesfamicael et al. summarized the efficacy and safety of PGx-guided antidepressant prescribing in patients with major depression (Tesfamicael et al.). Compared to patients getting treatment as usual, patients who received PGx-guided antidepressants had a 41%–78% higher chance of achieving remission and a 20%–49% higher chance of responding to antidepressants. However, their evaluation also revealed that the evidence regarding the effectiveness of PGx testing in depression varies greatly, with study design and the type of PGx test having a significant impact on outcomes. Furthermore, evaluating the effectiveness of PGx-guided treatment was the focus of most studies. The safety of PGx-guided antidepressant prescribing, however, is not well supported by the research to date. Lastly, there is scant or no data about the effects of PGx-guided antidepressant treatment on quality of life, recovery, relapse or suicide rates. Identifying which antidepressants benefit more from using PGx for better response would be a useful contribution for the regulatory and consortium agencies to conduct additional research and validations. This study builds on the numerous systematic reviews and meta-analyses published on this topic over the last few years, supporting the general conclusion that PGx-antidepressant prescribing improves the rates of response and remission, at least in the first few months of treatment (Brown et al., 2022).

Also regarding major depression, the minireview presented by Barlati et al. highlighted how, despite some encouraging results, several critical issues should be taken into account when considering PGx-guided antidepressant prescribing (Barlati et al.). Since significant changes of clinical relevance may occur during this time, and the duration of response and sustained remission represent aspects that should be carefully evaluated, it is important to analyse clinical outcomes after at least 8 weeks, which represents the typical duration of treatment of a depressive episode in the acute phase, and to extend the evaluation for 12 or 24 weeks. Moreover, the absence of prescriber does not preclude a potential influence on outcome assessment. Future research should use a more uniform approach to participant inclusion criteria and consider a further key factors that define the clinical definition of major depressive disorder. Comorbidities, substance and alcohol addiction, trauma history and a family history of psychiatric disorders are additional crucial factors. Most clinical studies predominantly enrol Caucasian females in middle age, and many studies have significant financial conflicts of interest**.** In conclusion, this minireview suggests that PGx testing may be beneficial in the management of major depression, nevertheless, it is still unclear if PGx can be applied routinely in clinical practice away from specialist centres who have considerable PGx experience.

Finally, Wang et al. aimed to evaluate the potential mechanisms of L-methionine’s inhibitory effects on cocaine induced cellular and behavioural changes (Wang et al.). They performed a MRNA and miRNA high-throughput sequencing of the prefrontal cortex in a mouse model of cocaine conditioned place preference combined with L-methionine. They identified differentially expressed miRNAs (DE-miRNAs) and differentially expressed genes (DEGs) regulated by cocaine and inhibited by L-methionine. Functional annotation of identified DEGs were evaluated. MiRNA-mRNA regulatory network and miRNA-mRNA-TF regulatory networks were established to screen key DE-miRNAs and coregulation network. Sequencing data analysis showed that L-methionine reversely regulated genes and miRNAs affected by cocaine. Pathways associated with drug addiction only enriched in CS-down with MC-up genes targeted by DE-miRNAs including GABAergic synapse, glutamatergic synapse, circadian entrainment, axon guidance and calcium signalling pathway. Drug addiction associated network was formed of 22 DEGs including calcium channel, ephrin and ryanodine receptor genes. The calcium channel gene network was identified as a core gene network modulated by L-methionine in response to cocaine dependence. Moreover, it was predicted that *Grin1* and *Fosb* presented in TF-miRNA-mRNA coregulation network with a high degree of interaction as hub genes and interacted calcium channels. These findings help better understand the underlying genetic mechanisms of L-methionine induced changes in cocaine addiction.

In conclusion, this Research Topic delves into interesting and timely topics about PGx in psychiatry, particularly the evolving clinical area of PGx-guided antidepressant prescribing and the complexities involved in understanding precisely the genetic mechanisms that occur during treatment with psychotropic medications e.g., pharmacodynamic changes. The collected works enrich our knowledge on the PGx of psychiatric disorders and highlight how difficult it is to implement new knowledge into clinical practice.
